# Numerical study of achiral phase-change metamaterials for ultrafast tuning of giant circular conversion dichroism

**DOI:** 10.1038/srep14666

**Published:** 2015-10-01

**Authors:** Tun Cao, Chenwei Wei, Libang Mao

**Affiliations:** 1 Department of Biomedical Engineering, Dalian University of Technology, 116024 China (P.R.C) 116024

## Abstract

Control of the polarization of light is highly desirable for detection of material’s chirality since biomolecules have vibrational modes in the optical region. Here, we report an ultrafast tuning of pronounced circular conversion dichroism (CCD) in the mid-infrared (M-IR) region, using an achiral phase change metamaterial (PCMM). Our structure consists of an array of Au squares separated from a continuous Au film by a phase change material (Ge_2_Sb_2_Te_5_) dielectric layer, where the Au square patches occupy the sites of a rectangular lattice. The extrinsically giant 2D chirality appears provided that the rectangular array of the Au squares is illuminated at an oblique incidence, and accomplishes a wide tunable wavelength range between 2664 and 3912 nm in the M-IR regime by switching between the amorphous and crystalline states of the Ge_2_Sb_2_Te_5_. A photothermal model is investigated to study the temporal variation of the temperature of the Ge_2_Sb_2_Te_5_ layer, and shows the advantage of fast transiting the phase of Ge_2_Sb_2_Te_5_ of 3.2 ns under an ultralow incident light intensity of 1.9 μW/μm^2^. Our design is straightforward to fabricate and will be a promising candidate for controlling electromagnetic (*EM*) wave in the optical region.

Structure has chirality if it does not superimpose onto its mirror image^1^. This geometric chirality produces a different response of right circularly polarized (RCP) and left circularly polarized (LCP) light, hence rotating the angle of linearly polarized propagating light. Chirality plays a crucial role in analytical chemistry and molecular nanoengineering^2^,^3^,^4^,^5^. Consequently, both intrinsically 3D-chiral effect i.e., optical activity and circular dichroism (CD)^6^,^7^, as well as 2D-chiral response i.e., asymmetric transmission and circular conversion dichroism (CCD)^8^,^9^ can be found in nature. In all natural chiral molecules though, chiral responses tend to be very weak and detectable only if strong phase differences between RCP and LCP waves accumulate over a long optical path^2^. To solve this problem, periodically plasmonic nanostructures known as chiral metamaterial (MM) and achiral MM have been recently proposed to obtain large chiral effects like optical activity^4^, asymmetric transmission^10^ and polarization conversion^11^,^12^,^13^,^14^,^15^. Particularly, chiral MM has two different mirror forms called enantiomers and non-chiral MM only breaks mirror symmetry under oblique incidence. Nonetheless, fabrication of intrinsically 3D chiral MM, such as helix^16^ and twisted bi-layer configuration^17^ in the high frequency region, is still tough. Therefore, very recently 2D planar chiral MM and achiral MM, also known as chiral metasurface^18^ and achiral metasurface^19^, have been proposed to achieve significant chirality in the optical region due to their reduced complexity of fabrication.

Despite achievement of strong chiral effect from MMs, recent progress in tunable MMs has led to the realization of dynamic control of circular polarization of EM waves, which is desirable for highly sensitive detection of biomolecule’s chirality as biomolecules have vibration modes. For instance, optical activity in MMs can be actively tuned through external stimulus like electrostatic actuation^20^, heat^21^ or photoexcitation^22^,^23^,^24^,^25^,^26^. The polarization phenomena can also be passively tuned by the tilt of the MM plane relative to the incident beam^27^,^28^ or changing the geometry of the resonance element^29^. Even so, tunable chirality from the MMs still suffers a slow switching speed and narrow tuning range, not to mention the great manufacturing complexity in the optical region. It is still a formidable challenge to ultrafast tune a giant 2D-chiral response with a wide tuning range in the optical region.

Here, we demonstrate that the resonant frequency of giant CCD can be tuned in the M-IR regime using an achiral phase change metamaterial (PCMM). The achiral PCMM is composed of an array of thin Au squares separated from a continuous Au film by an active dielectric interlayer of prototypical phase change material (PCM): Ge_2_Sb_2_Te_5_, where the Au squares occupy the sites of a rectangular lattice. This structure can be called ‘2D planar metamaterial’ since its planar reflectarray of Au squares exhibits subwavelength periodicity and negligible thicknesses as compared to the incident wavelength^30^.

The giant CCD analogous to chiral MMs can be achieved, provided that the structure is illuminated under oblique incidence. This large difference between the left-to-left and right-to-right circularly polarized reflectance conversion efficiencies so called as CCD, attributes to the strong electric and magnetic resonances in the multilayer MM^31^. It is because the chirality is characterized by electric and magnetic dipolar moments and enhancing chirality spectroscopy entails manipulation of both electric and magnetic fields of light^32^,^33^. With the integration of PCM, a broad range of the spectral tunability of CCD in the proposed structure can be obtained by switching between the amorphous and crystalline states of Ge_2_Sb_2_Te_5_. Importantly, we construct a heat model to show that the temperature of amorphous Ge_2_Sb_2_Te_5_ can be raised from room temperature to 433 K (amorphous-to-crystalline phase transition temperature)^34^,^35^ in just 3.2 ns with a low light intensity of 1.9 μW/μm^2^, owing to the enhanced light absorbance over a wide range of incident angles through strong plasmonic resonances in the structure^36^. Thereby, our structure can supply sufficient thermal energy to change the amorphous phase to crystalline phase for both LCP and RCP light sources.

We hope the results presented herein will serve as an impetus for the development of PCM specifically for achiral MMs exhibiting tunable 2D-chiroptical effect. Whilst, this tunable PCMM is relatively straightforward to fabricate. Interestingly, PCM does not require any energy to maintain the phase of the material. Therefore once the PCMM has been switched it will retain its chiral response until it is switched again. This clearly makes MM based on PCM interesting from a ‘green technology’ perspective. Finally, the proposed PCMM is ultrathin that may be integrated within today’s nanophotonic systems. It can find diverse functionalities, such as biomolecule sensing, switches, circular polarization transformers, and modulators.

## Results

**Figure 1:**
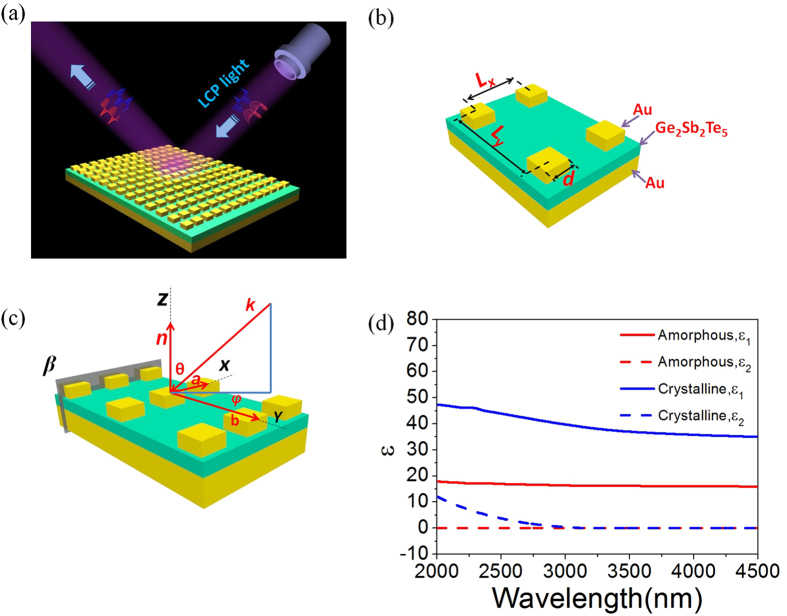
(**a**)Schematic of PCMM based on Ge_2_Sb_2_Te_5_ dielectric interlayer. The thicknesses of the Au squares, Ge_2_Sb_2_Te_5_ and Au mirror are 40, 40 and 80 nm, respectively. (**b**) Illustration of PCMM’s rectangular lattice pattern, where *L*_*x*_ = 400 nm, *L*_*y*_ = 800 nm and *d* = 200 nm. (**c**) Demonstration of *k*, *n*, *a*, *b*, *θ* and *φ*, marked in red.(**d**) Dielectric constant *ε*_1_(*ω*) and *ε*_2_(*ω*) vs wavelength for both amorphous and crystalline phases of Ge_2_Sb_2_Te_5_.

Figure 1(a) shows the proposed PCMM consisting of two Au layers spaced by a 40 nm thick Ge_2_Sb_2_Te_5_ dielectric interlayer. The top metal layer is a 40 nm thick Au square array, where the pitches along the *x* and *y* directions are *L*_*x*_ = 400 nm and *L*_*y*_ = 800 nm, respectively. The side length of the Au square is *d* = 200 nm. The bottom Au layer has a thickness of 80 nm, which will prevent the transmission of the incident light hence leading to a nearly zero transmittance^31^. Moreover, the strong electric and magnetic dipolar resonances in the multilayer structure may introduce a big difference in the absorbance (reflectance) for RCP and LCP waves under off-normal incidence. The scheme of the unit cell is shown in Fig. 1(b), while Fig. 1(c) shows the incident wavevector *k*, the vector normal to the surface *n*, and the two primitive lattice vectors (*a* and *b*) in red. The incident angle *θ* is measured between the wavevector *k* and the vector *n*. *φ* is the rotation angle between the parallel (to the *x–y* plane) component of *k* and the *x* axis. The structure is suspended in a vacuum. A simple Drude model is used for the dielectric constant of Au, 
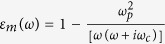
 where 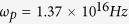 is the plasma frequency and 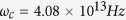 is the scattering frequency for Au^37^. The simulation is performed by commercial finite integration package CST MICROWAVE STUDIO®.

The real, *ε*_*1*_*(ω)* and imaginary, *ε*_*2*_*(ω)* parts of the dielectric function for the different states of Ge_2_Sb_2_Te_5_ were obtained from the published Fourier transform infrared spectroscopy data in^38^, which for the M-IR spectral range are shown in Fig. 1(d). A pronounced change in the *ε*_1_(ω) is obtained during the reversible structural transformation from amorphous to crystalline. The dielectric constant of Ge_2_Sb_2_Te_5_ is very dispersive and changes back to its initial value for the reversible structural transformation from amorphous to crystalline. These very different optical properties are realistic and well known but they have predominantly been applied to data storage applications. It should be mentioned that the reversible phase transition in Ge_2_Sb_2_Te_5_ is highly repeatable and more than a billion cycles have been experimentally demonstrated in data storage devices^39^. With these unique properties, the Ge-Sb-Te system is of great interest for actively tunable plasmonics and nanophotonics^40^,^41^.

**Figure 2:**
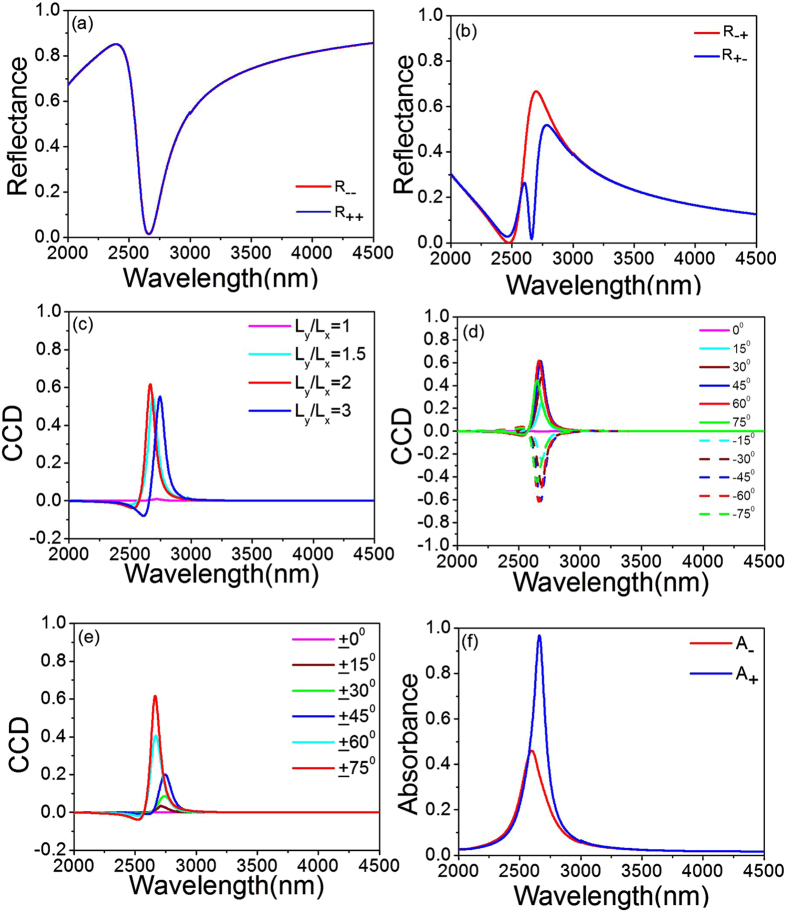
The spectra of (**a**) *R*_− −_ and *R*_++_;(**b**) 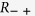 and 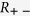 with *L*_*x*_ = 400 nm and *L*_*y*_ = 800 nm for amorphous state at *θ* = 75°, *φ* = 60°; (**c**) CCD for *θ* = 75°, *φ* = 60° at different *L*_*y*_*/L*_*x*_ with *L*_*x*_ = 400 nm and amorphous state; (**d**) CCD for *θ* = 75° with different *φ* at *L*_*x*_ = 400 nm, *L*_*y*_ = 800 nm and amorphous state; (**e**) CCD for *φ* = 60° with different *θ* at *L*_*x*_ = 400 nm, *L*_*y*_ = 800 nm and amorphous state; (**f**) the absorbance for RCP and LCP incident light for amorphous state with *θ* = 75°, *φ* = 60°, *L*_*x*_ = 400 nm and *L*_*y*_ = 800 nm.

In order to manifest chiroptical response, here the complex circular reflection matrix *r*_*ij*_ of the achiral PCMM is defined in terms of the incident 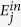 and reflected 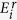 field vector by 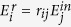, where ‘*i*’ and ‘*j*’ are the subscripts correspond to RCP (+) and LCP (−) components^42^. The reflectance is defined as 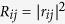 and presents the intensities of the corresponding reflected and converted components for RCP and LCP incident waves, and the total reflectance through the PCMM is expressed as 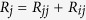. The diagonal terms *r*_++_ and *r*_− −_ of *r*_*ij*_ are used to detect 3D chirality which is linked to the circular dichroism (CD): 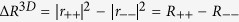, where *R*_++_ and *R*_− −_ stand for direct reflectance of circular polarization. Similarly, the off-diagonal terms *r*_−+_ and *r*_+−_ of *r*_*ij*_ represent the values demonstrating 2D chirality related to circular conversion dichroism (CCD): 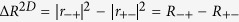, where *R*_−+_ stands for left-to-right polarized conversion efficiencies and *R*_+−_ for right-to-left polarized conversion efficiencies in reflectance^8^,^43^. To detect the extrinsic 3D-chiral response, Fig. 2(a) shows the diagonal elements of the reflectance matrix (*R*_− −_ and *R*_++_) in the amorphous state for LCP and RCP oblique incidence with *θ* = 75°, *φ* = 60°, *L*_*x*_ = 400 nm and *L*_*y*_ = 800 nm. As can be seen, the *R*_− −_ and *R*_++_ are equal (CD = 0) showing the absence of an extrinsic 3D chirality. Analogously, to detect the extrinsic 2D-chiral effect, we calculate the off-diagonal elements *R*_−+_ and *R*_+−_ of the reflectance matrix shown in Fig. 2(b). The spectra for *R*_−+_ and *R*_+−_ lie on top of each other away from the optimal wavelength 2664 nm, around which 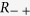 and 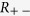 change sharply in opposite directions. At the optimal wavelength, a large difference between 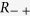 and 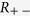 is linked to a giant CCD (2D-chiral effect) of 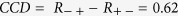, where 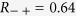 and 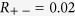. This CCD arises from the mutual orientation of non-chiral elements and the direction of the light propagation, and its significant value of 0.62 originates from the strong electric and magnetic resonances in the multilayer structure. It should be mentioned that although our structure has a nearly zero transmittance, one may alternatively utilize the reflectance spectra for the possible applications of the 2D chirality.

Figure 2(c) shows the CCD spectra of the amorphous structure with different ratios of *L*_*y*_*/L*_*x*_ at *L*_*x*_ = 400 nm for *θ* = 75°, *φ* = 60°. The 2D-chiral effect vanishes (CCD = 0) for a square array of the Au patches (*L*_*x*_ = *L*_*y*_ = 400 nm) as the plane of incidence falls on a line of mirror symmetry of the structure. By increasing the ratio of *L*_*y*_*/L*_*x*_, the CCD can be gradually improved. However, the CCD response is weakened when the ratio of L_y_/L_x_ is larger than 2 (*L*_*y*_*/L*_*x*_ > 2). It is because the coupling of neighboring Au squares along the *y* direction is reduced as increasing *L*_*y*_. Figure 2(d) demonstrates the CCD spectra for different values of *φ* with *θ* = 75° at *L*_*x*_ = 400 nm and *L*_*y*_ = 800 nm, where the CCD can achieve its maximum value of 0.62 at *φ* = 60°. Meanwhile, the 2D-chiroptical effects disappear for *φ* = 0° and 90° as the anisotropic axis of the structure is in the incident plane hence leading to a mirror plane of the experimental geometry. Moreover, opposite rotation angles (±*φ*) result in opposite signs of CCD corresponding to two enantiomeric arrangements.

We then study the effect of variation of incident angles on the CCD for fixed *φ* = 60° at *L*_*x*_ = 400 nm and *L*_*y*_ = 800 nm. As can be seen in Fig. 2(e), the CCD is varied by tilting the array of Au squares from *θ* = 0° to 75° and obtain the highest value of 0.62 at 2664 nm for *θ* = 75°. Therefore, the 2D-chiroptical response herein is optimized at *θ* = 75° and *φ* = 60°. Moreover, it should be noted that the spectra of CCD do not reverse when the signs of *θ* are opposite due to the property of extrinsic 2D chirality.

Figure 2(f) shows the absorbance for RCP (*A*_+_) and LCP (*A*_−_) incident light with *θ* = 75*°*, *φ* = 60°, *L*_*x*_ = 400 nm and *L*_*y*_ = 800 nm, where 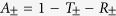 and the bottom Au layer prevents incident light transmitting through the top two layers hence leading to a nearly zero transmittance (*T*_±_ = 0). A maximum absorbance of 0.97 (*A*_+_ = 0.97) for RCP incidence is achieved at the resonance wavelength of 2664 nm, whereas the absorbance deceases to *A*_−_ = 0.35 for the LCP. The absorbance peaks originates from the electric and magnetic resonances in the achiral PCMM, which can in turn contribute to the strong absorbance difference (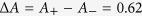).

**Figure 3:**
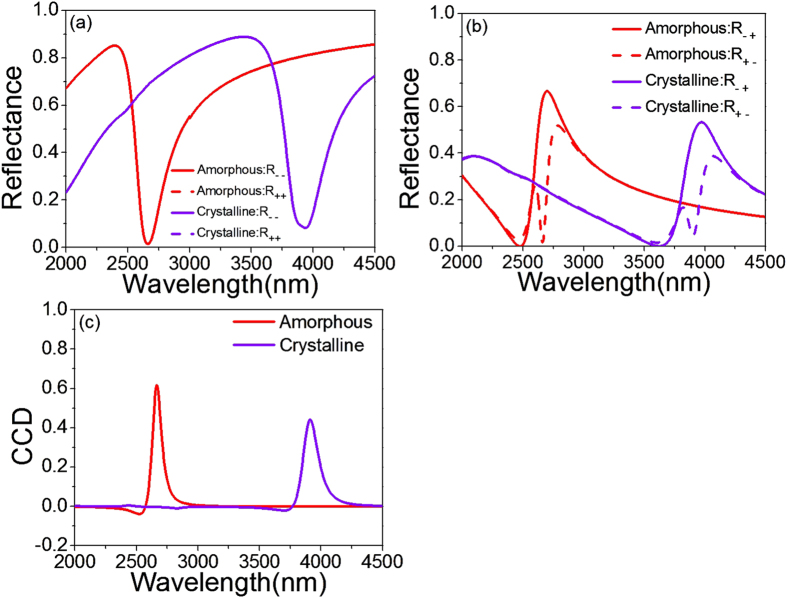
The spectra of (**a**) *R*_− −_ and *R*_++_;(**b**) 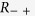 and 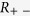; (**c**) The CCD spectra with *L*_*x*_ = 400 nm and *L*_*y*_ = 800 nm, at *θ* = 75°, *φ* = 60° in the amorphous and metastable cubic crystalline states.

The most interesting advantage of the achiral PCMM is their fast and broad tunability. Figure 3(a) shows the simulated diagonal elements of the reflectance matrix (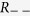 and 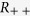) of the Au squares array with *L*_*x*_ = 400 nm and *L*_*y*_ = 800 nm for LCP and RCP illuminations at *θ* = 75°, *φ* = 60°, for the amorphous state and crystalline state Ge_2_Sb_2_Te_5_. It can be seen that 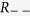 and 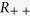 overlap (CD = 0) for both of the states, and the reflectance dip shifts towards longer wavelength (from 2664 to 3912 nm) when the phase of Ge_2_Sb_2_Te_5_ is switched from amorphous to crystalline, which is a 46% tuning range. In Fig. 3(b), we also find that the off-diagonal elements (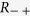 and 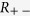) change sharply in opposite directions around 2664 nm for amorphous Ge_2_Sb_2_Te_5_ and 3912 nm for crystalline Ge_2_Sb_2_Te_5_, indicating large CCD at the resonant wavelengths for both of the sates. Figure 3(c) shows the evolution of the CCD in the reflectance spectra. With the phase change of Ge_2_Sb_2_Te_5_, the resonance wavelength of the CCD spectra at 2664 nm for the amorphous state shifts to the 3912 nm for crystalline state. Thanks to the large tuning range for the CCD shift of 1248 nm, this proposed PCMM can be very useful in switching on/off the 2D-chiral effect. For example, the CCD appears to be zero around 2664 nm, and thus switching off the chiroptical response when Ge_2_Sb_2_Te_5_ is transited from the amorphous to crystalline. We also find that the maximum value of CCD at 3912 nm decreases to 0.44 when Ge_2_Sb_2_Te_5_ changes from the amorphous to crystalline in addition to a shift, owing to the weaker magnetic resonance in the crystalline Ge_2_Sb_2_Te_5_.

**Figure 4:**
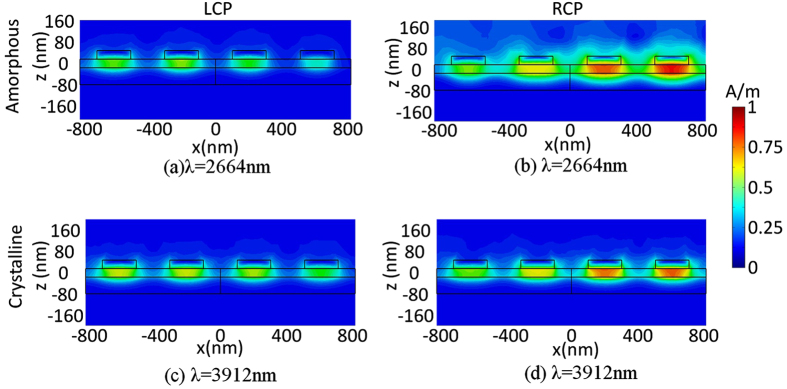
Map of the normalized total magnetic field intensity (*H*) distribution along the plane *β*: resonance wavelength at 2664 nm for amorphous Ge_2_Sb_2_Te_5_ under (a) LCP incidence, (b) RCP incidence with *θ* = 75°, *φ* = 60°; resonance wavelength at 3912 nm for crystalline Ge_2_Sb_2_Te_5_ under (c) LCP incidence, (d) RCP incidence with *θ* = 75°, *φ* = 60°.

The giant CCD results from the coupling between the Au- Ge_2_Sb_2_Te_5_-Au multilayers, which gives rise to a strong magnetic response. This strong magnetic resonance is connected to an antisymmetric charge-oscillation eigenmode, providing the combined plasmon mode a twist in the propagation direction of the wave to imitate 2D chirality^44^. In Fig. 4, we show the total magnetic field intensity 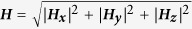 distributions at *β* plane shown in Fig. 1(c), associated with the resonant wavelength of 2664 nm for the amorphous and 3912 nm for the crystalline Ge_2_Sb_2_Te_5_ induced by the off-normal LCP and RCP incident lights at *θ* = 75°, *φ* = 60°. Figure 4(a,b) show that the *H* fields in the amorphous phase can be efficiently confined in the Ge_2_Sb_2_Te_5_ layer between the Au layers, which is owing to a concomitant coupling between surface plasmons counterpropagating on the two closely spaced interfaces^45^. Meanwhile, the *H* field distributions in the crystalline phase shown in Fig. 4(c,d) are similar to the amorphous phase shown in Fig. 4(a,b) for both LCP and RCP incidences respectively, which implies that the magnetic resonant dipole can also be excited to create CCD in the crystalline state. However, the difference of the *H* fields of the crystalline Ge_2_Sb_2_Te_5_ between the RCP and LCP incidences is smaller than that of the amorphous Ge_2_Sb_2_Te_5_ hence leading to a smaller CCD shown in Fig. 3(c). As can be seen in Fig. 4, the *H* field pattern appears asymmetric with respect to center under the oblique incidence of θ = 75°, φ = 60°. This is because the time of pulse propagating through different regions of the structure is unequal. This also explains how the oblique incidence causes the asymmetric reflectance of the two circular polarization lights shown in Fig. 2(a,b).

Since the reversible amorphous - crystalline phase transition of Ge_2_Sb_2_Te_5_ can be induced through optical heating, it is important to understand the heat induced switching behavior of the achiral PCMM. To show this, a heat transfer model developed from our previous work^36^ is further investigated to obtain the temporal variation of temperature of Ge_2_Sb_2_Te_5_ layer for different polarized incident light using the Finite Element Method (FEM) solver within COMSOL. Here, the achiral PCMM model is set up in COMSOL identical to that shown in Fig. 1. A Gaussian pulse is used as the excitation source to evaluate the required time to switch from the amorphous to the crystalline state of the Ge_2_Sb_2_Te_5_. In particular, the Gaussian source has a repetition rate *f*_*r*_ = 25 kHz and pulse duration of 2.6 ns. The light fluence shining on the sample from a single pulse is written as 
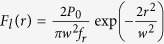
36, where *P*_*0*_ = 0.6 mW is the total incident power, *r* is the distance from the beam center, *w* = 10 μm is Gaussian beam waist.

**Figure 5:**
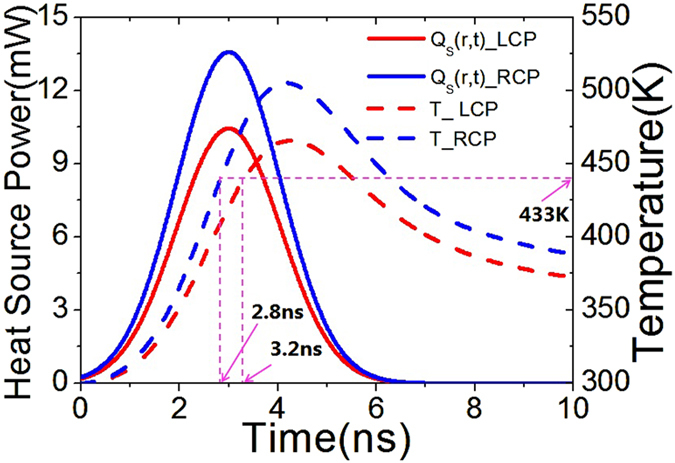
3D- FEM simulation of heat power irradiating on an achiral PCMM located at the beam center, where the solid red line presents the heat power irradiating on the structures for LCP incident light, the solid blue line presents the heat power irradiating on the structures for RCP incident light, the dash red line is the temperature of the amorphous Ge_2_Sb_2_Te_5_ layer during one pulse for LCP incident light, the dash blue line is the temperature of amorphous Ge_2_Sb_2_Te_5_ layer during one pulse for RCP incidence.

The thermal conductivity of Ge_2_Sb_2_Te_5_ changes with the temperature are obtained from experiment data in^46^. The thermal energy absorbed by one unit cell is expressed as 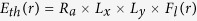 where *L*_*x*_ = 400 nm, *L*_*y*_ = 800 nm and *R*_*a*_ the absorbance coefficient of the absorber is 0.1 for LCP and 0.14 for RCP incident light respectively, derived from the overlap integral between the light source power density spectra and the absorbance spectra for *θ* = 75°, *φ* = 60°, shown in Fig. 2(f). The heat source power is expressed by a Gaussian pulse function


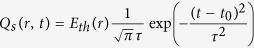


where *τ* = 1.5 ns is the time constant of the light pulse, *t*_*0*_ = 3 ns is the time delay of the pulse peak. Figure 5 shows *Q*_*s*_*(r, t)* and the temperature of the amorphous Ge_2_Sb_2_Te_5_ interlayer for both LCP and RCP incidences at *θ* = 75°, *φ* = 60°, where the PCMM is placed at the center of Gaussian beam. As can be seen, the temperature of amorphous Ge_2_Sb_2_Te_5_ for the RCP can reach 433 K at 2.8 ns and maximum 505 K at 4.2 ns under an incident light intensity of 1.9 μW/μm^2^. Due to heat dissipation to the surroundings, the temperature starts decreasing after 4.2 ns before the next pulse comes. However, *Q*_*s*_(*r*, *t*) and the temperature for the LCP are lower than the RCP owing to its smaller absorbance coefficient *R*_*a*_, where the amorphous-to-crystalline phase transition temperature of 433 K is achieved at 3.2 ns under the same light intensity of 1.9 μW/μm^2^. Thereby the melting point of 433 K can be obtained to switch the phase of Ge_2_Sb_2_Te_5_ for both LCP and RCP incident light at *θ* = 75°, *φ* = 60°.

Tuning of chiroptical response at a large power input may sound challenging, since the high power illumination gives rise to unwanted heating to prevent polarization tuning. To solve this problem, herein an Au-Ge_2_Sb_2_Te_5_-Au trilayers structure is proposed, where the Au bottom layer will interact with the upper Au patches to form electric and magnetic dipoles that efficiently couple the incident light into the dielectric interlayer of Ge_2_Sb_2_Te_5_^31^. It has the advantage of transiting the phase of Ge_2_Sb_2_Te_5_ under RCP and LCP illumination with an ultralow light intensity of 1.9 μW/μm^2^ and thus may not prevent the polarization tuning. Meanwhile, we suggest that a future design, with the second external pumping laser, of weaker intensity, should be introduced to heat up the Ge_2_Sb_2_Te_5_ layer to change the phase. Or alternatively, one may apply a voltage between the top layer Au patches and bottom Au mirror to electrically switch the state of Ge_2_Sb_2_Te_5_. By means of the external stimulus, the phase transition of Ge_2_Sb_2_Te_5_ can be carried out separately hence not interacting with the polarization tuning.

## Discussion

In conclusion, we have demonstrated an ultrafast tuning of giant CCD using an achiral PCMM and a large frequency shift of 46% for CCD is observed in the M-IR regime. This CCD is caused by the mutual orientation of the achiral PCMM and the oblique incident wave, where the giant value of the CCD is due to the strong magnetic dipolar resonance in the multilayer structure. The ultrafast tunable effect is due to the short time (3.2 ns) of phase transition from the amorphous to crystalline in the structure under a low pump light intensity (1.9 μW/μm^2^). This work presents a new method to massively tune the resonant frequency of giant CCD in an achiral metamaterial, and can find numerous applications in ultrathin polarization rotators, modulators and circular polarizers.

## Methods

For the numerical calculations of the CCD spectra, we used the commercial finite integration package CST MICROWAVE STUDIO®. To account for the periodic nature of the structure, the model boundary in the *x* and *y* directions is set to unit cell boundary conditions, respectively. To achieve the temporal variation of temperature of Ge_2_Sb_2_Te_5_ layer, we used the Finite Element Method by means of COMSOL Multiphysics: the model boundary at 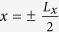 and 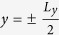 is set to condition of perfect magnetic conductor and perfect electric conductor, respectively. A simple Drude model is used for the dielectric constant of Au, 
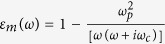
 where 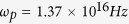 is the plasma frequency and 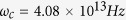 is the scattering frequency for Au^37^. The dielectric constants for the different states of Ge_2_Sb_2_Te_5_ were obtained from the published Fourier transform infrared spectroscopy data in^38^.

## Additional Information

**How to cite this article**: Cao, T. *et al.* Numerical study of achiral phase-change metamaterials for ultrafast tuning of giant circular conversion dichroism. *Sci. Rep.* **5**, 14666; doi: 10.1038/srep14666 (2015).

## References

[b1] Kelvin L. Baltimore Lectures on Molecular Dynamics and the Wave Theory of Light. Clay and Sons (London, pp 449) (1904).

[b2] Shaltout A., Liu J., Shalaev V. M. & Kildishev A. V. Optically Active Metasurface with Non-Chiral Plasmonic Nanoantennas. Nano Lett. 14, 4426–4431 (2014). 25051158 10.1021/nl501396d

[b3] He Y., Larsen G. K., Ingram W. & Zhao Y. Tunable Three-Dimensional Helically Stacked Plasmonic Layers on Nanosphere Monolayers. Nano Lett. 14, 1976–1981 (2014). 24646023 10.1021/nl404823z

[b4] Valev V. K., Baumberg J. J., Sibilia C. & Verbiest T. Chirality and Chiroptical Effects in Plasmonic Nanostructures: Fundamentals, Recent Progress, and Outlook. Adv. Mater. 25, 2517–2534 (2013). 23553650 10.1002/adma.201205178

[b5] Cui Y., Kang L., Lan S., Rodrigues S. & Cai W. Giant Chiral Optical Response from a Twisted-Arc Metamaterial. Nano Lett. 14, 1021–1025 (2014). 24422639 10.1021/nl404572u

[b6] Lindell I. V., Sihvola A. H., Tretyakov S. A. & Viitanen A. J. Electromagnetic Waves in Chiral and Bi-isotropic Media. Artech House (Boston, London, 1994).

[b7] Ranjbar B. & Gill P. Circular Dichroism Techniques: Biomolecular and Nanostructural Analyses- A Review. Chem. Biol. Drug Des. 74, 101–120 (2009). 19566697 10.1111/j.1747-0285.2009.00847.x

[b8] Plum E., Fedotov V. A. & Zheludev N. I. Extrinsic electromagnetic chirality in metamaterials. J. Opt. A: Pure Appl. Opt. 11, 074009 (2009).

[b9] Fullman R. L. & Wood D. L. Origin of spiral eutectic structures L’origine des structures eutectiques, spiraliformes Der ursprung der eutektischen spiralstrukturen. Acta Metall. 2, 188–189 (1954).

[b10] Schwanecke A. S. *et al.* Nanostructured Metal Film with Asymmetric Optical Transmission. Nano Lett. 8, 2940–2943 (2008). 18720979 10.1021/nl801794d

[b11] Feng L. *et al.* Metamaterials for Enhanced Polarization Conversion in Plasmonic Excitation. ACS Nano 5, 5100–5106 (2011). 21500845 10.1021/nn201181p

[b12] Feng L., Liu Z. & Fainman Y. Direct observation of plasmonic index ellipsoids on a deep-subwavelength metallic grating. Appl. Optics 50, G1–6 (2011). 10.1364/AO.50.0000G1 22086031

[b13] Katsa M. A. *et al.* Giant birefringence in optical antenna arrays with widely tailorable optical anisotropy. P. Natl. Acad. Sci. USA 109, 12364–12368 (2012).

[b14] Tremain B., Rance H. J., Hibbins A. P. & Sambles J. R. Polarization conversion from a thin cavity array in the microwave regime. Sci. Rep. 5, 9366 (2015). 25797210 10.1038/srep09366 PMC4369744

[b15] Cao T., Wei C. W., Mao L. B. & Li Y. Extrinsic 2D chirality: giant circular conversion dichroism from a metal-dielectric-metal square array. Sci. Rep. 4, 7442 (2014). 25501766 10.1038/srep07442 PMC4262820

[b16] Gansel J. K. *et al.* Gold Helix Photonic Metamaterial as Broadband Circular Polarizer. Science 325, 1513–1515 (2009). 19696310 10.1126/science.1177031

[b17] Cao T., Zhang L., Simpson R. E., Wei C. W. & Cryan M. J. Strongly tunable circular dichroism in gammadion chiral phase-change metamaterials. Opt. Express 21, 27841–27851 (2013). 24514301 10.1364/OE.21.027841

[b18] Jia Y. P. *et al.* Complementary chiral metasurface with strong broadband optical activity and enhanced transmission. Appl. Phys. Lett. 104, 011108 (2014).

[b19] Wang F., Wang Z. & Shi J. Theoretical study of high-Q Fano resonance and extrinsic chirality in an ultrathin Babinet-inverted metasurface. J. Appl. Phys. 116, 153506 (2014).

[b20] Kan T. *et al.* Spiral metamaterial for active tuning of optical activity. Appl. Phys. Lett. 102, 221906 (2013).

[b21] Cui L., Huang Y., Wang J. & Zhu K.-Y. Ultrafast modulation of near-field heat transfer with tunable metamaterials. Appl. Phys. Lett. 102, 053106 (2013).

[b22] Zhang S. *et al.* Photoinduced handedness switching in terahertz chiral metamolecules. Nat. Commun. 3, 942 (2012). 22781755 10.1038/ncomms1908

[b23] Zhou J. *et al.* Terahertz chiral metamaterials with giant and dynamically tunable optical activity. Phys. Rev. B 86, 035448 (2012).

[b24] Kenanakis G. *et al.* Optically controllable THz chiral metamaterials. Opt. Express 22, 12149–12159 (2014). 24921336 10.1364/OE.22.012149

[b25] Lv T. T. *et al.* Optically controlled background-free terahertz switching in chiral metamaterial. Opt. Lett. 39, 3066–3069 (2014). 24978275 10.1364/OL.39.003066

[b26] Kanda N., Konishi K. & Kuwata-Gonokami M. All-photoinduced terahertz optical activity. Opt. Lett. 39, 3274–3277 (2014). 24876031 10.1364/OL.39.003274

[b27] Singh R., Plum E., Zhang W. & Zheludev N. I. Highly tunable optical activity in planar achiral terahertz metamaterials. Opt. Express 18, 13425–13430 (2010). 20588473 10.1364/OE.18.013425

[b28] Shi J. H., Zhu Z., Ma H. F., Jiang W. X. & Cui T. J. Tunable symmetric and asymmetric resonances in an asymmetrical split-ring metamaterial. J. Appl. Phys. 112, 073522 (2012).

[b29] Liu X. *et al.* Manipulating wave polarization by twisted plasmonic metamaterials. Opt. Mater. Express 4, 1003–1010 (2014).

[b30] Lapine M. & Tretyakov S. Contemporary notes on metamaterials. IET Microw. Antenna. P. 1, 307 (2007).

[b31] Landy N. I., Sajuyigbe S., Mock J. J., Smith D. R. & Padilla W. J. Perfect Metamaterial Absorber. Phys. Rev. Lett. 100, 207402 (2008). 18518577 10.1103/PhysRevLett.100.207402

[b32] Tang Y. & Cohen A. E., Enhanced Enantioselectivity in Excitation of Chiral Molecules by Superchiral Light. Science 332, 333–336 (2011). 21493854 10.1126/science.1202817

[b33] García-Etxarri A. & Dionne J. A. Surface-enhanced circular dichroism spectroscopy mediated by nonchiral nanoantennas. Phys. Rev. B 87, 235409 (2013).

[b34] Michel A.-K. U. *et al.* Using Low-Loss Phase-Change Materials for Mid-Infrared Antenna Resonance Tuning. Nano. Lett. 13, 3470–3475 (2013). 23742151 10.1021/nl4006194

[b35] Orava J. *et al.* Optical properties and phase change transition in Ge_2_Sb_2_Te_5_ flash evaporated thin films studied by temperature dependent spectroscopic ellipsometry. J. Appl. Phys. 104, 043523 (2008).

[b36] Cao T., Wei C. W., Simpson R. E., Zhang L. & Cryan M. J. Rapid phase transition of a phase-change metamaterial perfect absorber. Opt. Mate. Express 3, 1101–1110 (2013).

[b37] Zhang S. *et al.* Experimental Demonstration of Near-Infrared Negative-Index Metamaterials. Phys. Rev. Lett. 95, 137404 (2005). 16197179 10.1103/PhysRevLett.95.137404

[b38] Shportko K. *et al.* Resonant bonding in crystalline phase-change materials. Nat. Mater. 7, 653–658 (2008). 18622406 10.1038/nmat2226

[b39] Simpson R. E. *et al.* Interfacial Phase-Change Memory. Nat. Nanotech. 6, 501–505 (2011). 10.1038/nnano.2011.96 21725305

[b40] Cao T., Simpson R. E. & Cryan M. J. Study of tunable negative index metamaterials based on phase-change materials. J. Opt. Soc. Am. B 30, 439–444 (2013).

[b41] Michel A.-K. U. *et al.* Reversible Optical Switching of Infrared Antenna Resonances with Ultrathin Phase-Change Layers Using Femtosecond Laser Pulses. ACS Photonics 1, 833–839 (2014).

[b42] Plum E., Fedotov V. A. & Zheludev N. I. Planar metamaterial with transmission and reflection that depend on the direction of incidence. Appl. Phys. Lett. 94, 131901 (2009).

[b43] Plum E. *et al.* Metamaterials: optical activity without chirality. Phys. Rev. Lett. 102, 113902 (2009). 19392202 10.1103/PhysRevLett.102.113902

[b44] Decker M., Klein M. W., Wegener M. & Linden S. Circular dichroism of planar chiral magnetic metamaterials. Opt. Lett. 32, 856–858 (2007). 17339960 10.1364/ol.32.000856

[b45] Dayal G. & Ramakrishna S. A. Design of highly absorbing metamaterials for Infrared frequencies. Opt. Express 20, 17503–17508 (2012). 23038303 10.1364/OE.20.017503

[b46] Kuwahara M. *et al.* Measurement of the thermal conductivity of nanometer scale thin films by thermoreflectance phenomenon. Microelectron. Eng. 84, 1792 (2007).

